# Supplementary material for: Women’s Depressive Symptoms during the COVID-19 Pandemic: The Role of Pregnancy
Source: Int J Environ Res Public Health. 2021 Apr 18;18(8):4298. doi: 10.3390/ijerph18084298 (PMC8072624; doi:10.3390/ijerph18084298)
Supplement: Supplementary file 1 [file ijerph-18-04298-s001.zip › ijerph-1161114-supplementary.pdf]

Table S1. Pearson correlations of study variables among pregnant and non-pregnant women.

Title	1.	2.	3.	4.	5.	6.	7.	8.	9.	10.	11.
1. Age	1.00										
2. Education	.31***	1.00									
3. Married	-.09**	.07**	1.00								
4. Employment	-.01	-.19***	-.03	1.00							
5. Income	-.22***	-.21***	-.18***	.15***	1.00						
6. Children under 18	.44***	.10***	.13***	.02	-.02	1.00					
7. COVID-19 contact	-.02	.02	.03	-.01	-.02	.03	1.00				
8. COVID-19 undiagnosed	-.02	-.03	-.003	.03	.02	-.001	.09**	1.00			
9. Outdoor	-.03	-.006	-.06*	.02	.07**	-.004	-.02	.02	1.00		
10. Partner support	-.20***	.07*	.12***	-.10**	-.07**	-.23***	.03	-.07*	-.01	1.00	
11. Family/Friends support	-.16***	.02	.03	-.04	-.03	-.23***	.01	-.03	-.05*	.31***	1.00
12. Healthy behaviors	-.06*	.05	-.02	-.06	-.10***	-.31***	.02	-.04	-.09***	.24***	.20***

* $p < 0.05$, ** $p < 0.01$, *** $p < 0.001$.

Table S2. Pearson correlations of study variables among pregnant women.

Title	1.	2.	3.	4.	5.	6.	7.	8.	9.	10.	11.	12.	13.	14.	15.	16.	17.
1. Age	1.00																
2. Education	.30***	1.00															
3. Married	-.09**	**.07*	1.00														
4. Employment	-.006	***9-.1	03-.	1.00													
5. Income	-.22***	***1-.2	***18-.	.16***	1.00												
6. Children under 18	.44***	***10.	***13.	.02	-.002	1.00											
7. COVID-19 contact	-.02	2.0	3.0	-.01	-0.02	.03	1.00										
8. COVID-19 undiagnosed	2-.0	3-.0	3.00-	-.03	.02	.001	.09**	1.00									
9. Outdoor	3-.0	6-.00	-.06*	.02	.07**	-.004	-.02	.02	1.00								
10. Partner support	***20-.	*7.0	.12***	0**1-.	-.07**	-.23***	3.0	.07*	-.01	1.00							
11. Family/Friends support	***6-.1	2.0	3-.0	-.04	-.03	-.23***	01-.	-.03	-.05*	.30***	1.00						
12. Healthy behaviors	6*-.0	5.0	2-.0	-.06	-.09***	-.31***	.02	-.04	-.10***	.24***	.20***	1.00					
13. Gestational age (weeks)	4.00	01.0	-.006	-.01	-.02	-.02	.000	-0.004	.02	.05	.04	.01	1.00				
14. Prenatal appointment altered/canceled	-.08**	6-.0	.04	.07	.07*	.07*	.07*	.05	.11***	-.02	.01	-.05	.07*	1.00			
15. High risk Pregnancy	***4.1	.01-	.007	.04	.07*	.08**	-.05*	-.07*	.07*	-.08*	-.07*	-.05	-.02	-.09**	1.00		
16. Preparedness stress	-.20***	***0-.1	.07*	.03	.15***	-.06	.04	.10**	.14***	-.004	-.03	-.17***	.10***	.23***	.05	1.00	
17. Infection stress	-.13***	**-.09	.06*	.01	.12***	-.01	-.02	.07*	.14**	-.02	-.009	-.13***	.06	.21***	.09**	.70***	1.00
18. Positive Appraisal	-.17***	-.18***	-.05	.02	.16***	*-.12**	.01	-.001	.11***	.001	.04	.03	-.006	**9.0	2.0	.31***	***9.2

* $p < 0.05$, ** $p < 0.01$, *** $p < 0.001$.

מחשבות לגבי היריון בתקופת הקורונה

מגפת הקורונה העולמית עלולה להוביל לסוגים שונים של מחשבות ותחושות במהלך ההיריון. אנא קראי את המשפטים הבאים ודרגי אותם על הסקאלה שבין 1=מעט מאוד עד 5=הרבה מאוד

אני חוששת ללכת למעקב היריון בשל מגפת הקורונה	1	2	3	4	5
אני מוטרדת מכך שלא אקבל את הטיפול שאני זקוקה לו בהיריון בשל מגפת הקורונה	1	2	3	4	5
אני דואגת שאדבק בנגיף הקורונה כשאגיע לבית החולים כדי ללדת	1	2	3	4	5
אני דואגת שהתינוק שלי ידבק בנגיף הקורונה בבית החולים לאחר שיוולד	1	2	3	4	5
אני מרגישה שמגפת הקורונה עוזרת לי להעריך את ההיריון שלי יותר	1	2	3	4	5
אני דואגת מכך שלא יוכל להיות איתי מישהו במהלך הלידה	1	2	3	4	5
אני דואגת מכך שלא אהיה מוכנה ללידה בגלל ההגבלות שקשורות למגפת הקורונה	1	2	3	4	5
אני מרגישה שעצם היותי בהיריון מחזק אותי בתקופת המגפה	1	2	3	4	5
אני מוטרדת מכך שאני לא אוכלת מספיק בריא, ישנה מספיק או עושה מספיק פעילות גופנית בשל ההגבלות שקשורות למגפת הקורונה	1	2	3	4	5
אני מוטרדת מכך שמחלת הקורונה תפגע בתינוק/ת שלי	1	2	3	4	5
אני מוטרדת מכך שמחלת הקורונה תפגע בהיריון שלי (כמו הפלה או ללידה מוקדמת)	1	2	3	4	5
אני מודאגת מכך שהמגפה יכולה להרוס לי את תוכניות הלידה	1	2	3	4	5
אני מוטרדת מכך שיפרידו אותי מהתינוק/ת שלי לאחר הלידה בשל המגפה	1	2	3	4	5
אני חושבת על התינוק שיהיה לי כדי לעזור לעצמי לעבור זמנים קשים בתקופה המגפה	1	2	3	4	5
אני מוטרדת מכך שאנשים לא יוכלו לעזור לי לטפל בתינוק/ת שלי אחרי הלידה	1	2	3	4	5

Figure S1. COVID-19 Related Thoughts in Pregnancy (Preis, Mahaffey, and Lobel, 2020).

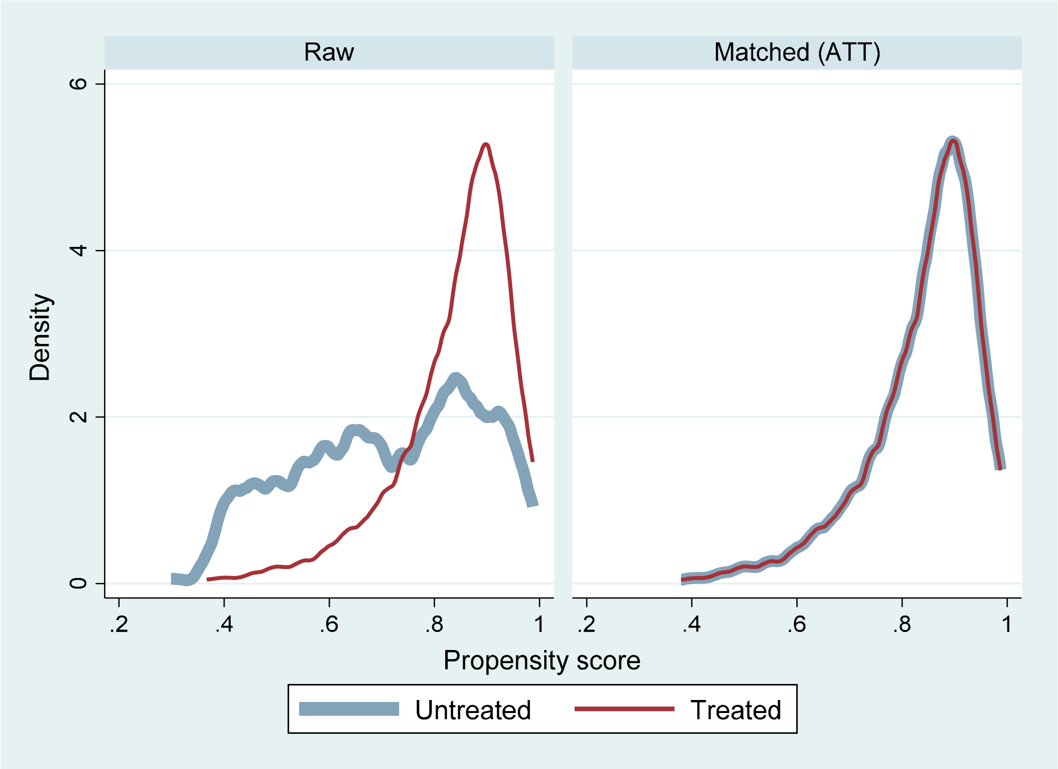

Figure S2. Propensity-score matching using a combination of age and education—evaluating balancing of the data.

Note: For post-estimation evaluation of data balancing, kernel density estimates before and after matching (y-axis) are displayed across propensity scores (x-axis). Based on regression-adjusted propensity-score kernel matching with age and education as covariates with a logit model, where pregnancy was defined as Treatment. ATT, average treatment effect on treated. Derived using KMATCH, Stata's module for multivariate-distance and propensity-score matching.

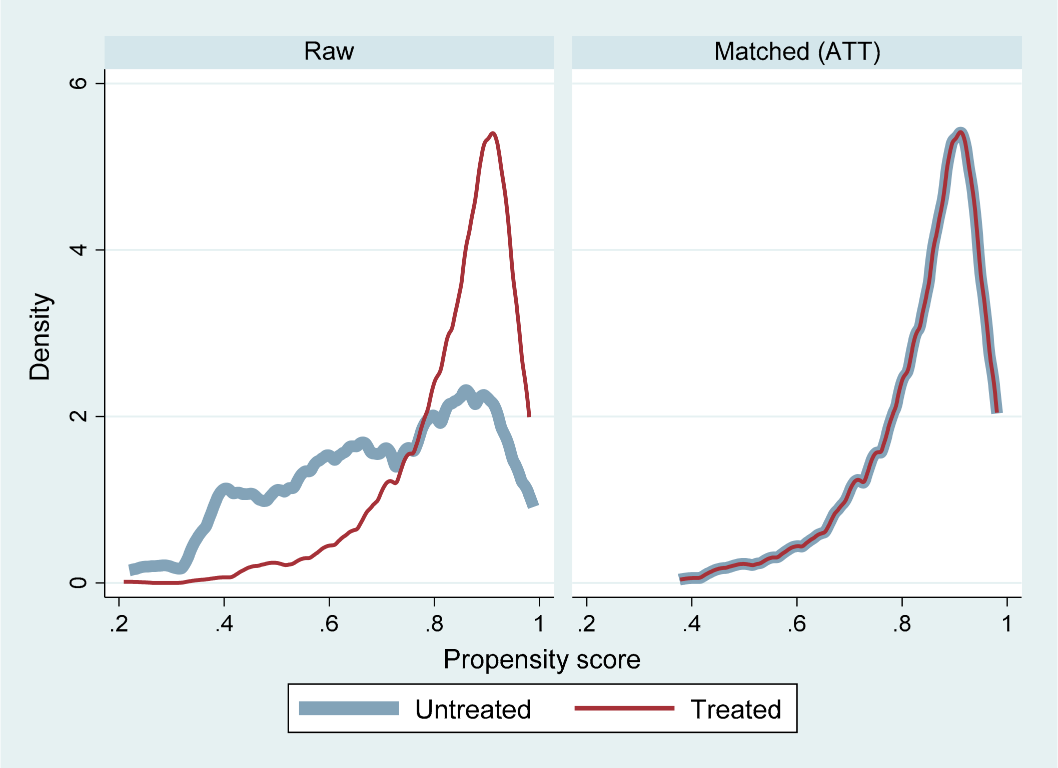

Figure S3. Propensity-score matching using a combination of age, education, income and employment status—evaluating balancing of the data.

Note: For post-estimation evaluation of data balancing, kernel density estimates before and after matching (y-axis) are displayed across propensity scores (x-axis). Based on regression-adjusted propensity-score kernel matching with age, education, income and employment status as covariates with a logit model, where pregnancy was defined as Treatment. ATT, average treatment effect on treated. Derived using KMATCH, Stata's module for multivariate-distance and propensity-score matching.

Table S3. Propensity-score matching using a combination of age and education—Stata output.

```
Propensity-score kernel matching                Number of obs     =      1,368
                                                Kernel            =       epan

Treatment   : preg = 1
Covariates  : age edu
PS model    : logit (pr)
RA equations: phq = age edu _cons
```

Matching statistics

	Matched			Controls			Bandwidth
	Yes	No	Total	Used	Unused	Total	
Treated	1103	9	1112	251	5	256	.0062601

Treatment-effects estimation

phq	Coef.	Std. Err.	t	P>|t|	[95% Conf.	Interval]
ATT	-.3070851	.1450836	-2.12	0.034	-.5916957	-.0224745
NATE	-.2182723	.1063224	-2.05	0.040	-.426845	-.0096995

Note: Regression-adjusted propensity-score kernel matching with age and education as covariates, based on a logit model where pregnancy was defined as Treatment. Nine pregnant women could not be matched, and five controls (non-pregnant women) were unused. Estimated effect of pregnancy in pregnant women following PS-matching (ATT) was similar to non-matched effects (NATE). ATT, average treatment effect on treated; NATE, non-matched average treatment effect. Based on KMATCH, Stata's module for multivariate-distance and propensity-score matching.

Table S4. Propensity-score matching using a combination of age, education, income and employment status—Stata output.

```
Propensity-score kernel matching                Number of obs     =      1,124
                                                Kernel            =       epan

Treatment   : preg = 1
Covariates  : age edu i.inc i.emp
PS model    : logit (pr)
RA equations: phq = age edu i.inc i.emp _cons
```

Matching statistics

	Matched			Controls			Bandwidth
	Yes	No	Total	Used	Unused	Total	
Treated	887	24	911	199	14	213	.0040486

Treatment-effects estimation

phq	Coef.	Std. Err.	t	P>|t|	[95% Conf.	Interval]
ATT	-.3104609	.152122	-2.04	0.041	-.6089361	-.0119856
NATE	-.2833496	.1181414	-2.40	0.017	-.5151524	-.0515468

Note: Regression-adjusted propensity-score kernel matching with age, education, income and employment status as covariates, based on a logit model where pregnancy was defined as Treatment. A total of 24 pregnant women could not be matched, and 14 controls (non-pregnant women) were unused. Estimated effect of pregnancy in pregnant women following PS-matching (ATT) was similar to non-matched effects (NATE). ATT, average treatment effect on treated; NATE, non-matched average treatment effect. Based on KMATCH, Stata's module for multivariate-distance and propensity-score matching.
